# Enhancing the radionuclide theranostic concept through the radiohybrid approach

**DOI:** 10.1039/d4md00591k

**Published:** 2024-11-25

**Authors:** Tobias Krönke, Klaus Kopka, Constantin Mamat

**Affiliations:** a Helmholtz-Zentrum Dresden-Rossendorf, Institute of Radiopharmaceutical Cancer Research Bautzner Landstraße 400 D-01328 Dresden Germany c.mamat@hzdr.de; b TU Dresden, Faculty of Chemistry and Food Chemistry D-01062 Dresden Germany; c National Center for Tumor Diseases (NCT) Dresden, University Hospital Carl Gustav Carus Fetscherstraße 74 D-01307 Dresden Germany; d German Cancer Consortium (DKTK) Partner Site Dresden, Fetscherstraße 74 D-01307 Dresden Germany

## Abstract

Radionuclide theranostics – a fast-growing emerging field in radiopharmaceutical sciences and nuclear medicine – offers a personalised and precised treatment approach by combining diagnosis with specific and selective targeted endoradiotherapy. This concept is based on the application of the same molecule, labelled with radionuclides possessing complementary imaging and therapeutic properties, respectively. In radionuclide theranostics, radionuclide pairs consisting of the same element, such as ^61/64^Cu/^67^Cu, ^203^Pb/^212^Pb or ^123/124^I/^131^I are of significant interest due to their identical chemical and pharmacological characteristics. However, such “true matched pairs” are seldom, necessitating the use of complementary radionuclides from different elements for diagnostics and endoradiotherapy with similar chemical characteristics, such as ^99m^Tc/^186/188^Re, ^68^Ga/^177^Lu or ^68^Ga/^225^Ac. Corresponding combinations of such two radionuclides in one and the same radioconjugate is referred to as a “matched pair”. Notably, the pharmacological behavior remains consistent across both diagnostic and therapeutic applications with “true matched pairs”, which may differ for “matched pairs”. As “true matched pairs” of theranostic radioisotopes are rare and that some relevant radionuclides do not fit with the diagnostic or therapeutic counterpart, the radionuclide theranostic concept can be expanded and improved by the introduction of the radiohybrid approach. Radiohybrid (rh) ligands represent a new class of radiopharmaceutical bearing two different positions for the introduction of a (radio)metal and (radio)halogen in one molecule, which can be then used for both therapeutic and diagnostic purposes. The following review will give an insight into recent developments of this approach.

## Abstract 2

The radiohybrid concept: a significant extension of radionuclide theranostics – a fast-growing field in radiopharmaceutical sciences offering personalised and precise treatment options by combining specific diagnosis with targeted endoradiotherapy.

## Graphical abstract

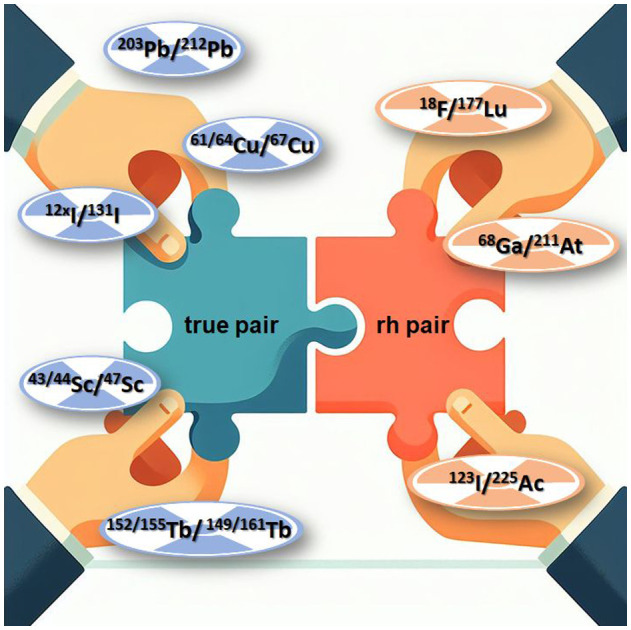

## Introduction

The combination of two binding sites in the same molecule for the binding of different radionuclides for either therapeutic or diagnostic applications is called the radiohybrid approach within the theranostic concept. Such radiopharmaceuticals bearing these two different radiolabelling positions are called radiohybrid (rh) ligands.^1^ Using this concept, a consistent pharmacological behaviour of the resulting radioconjugate is achieved. In contrast to the well-known radionuclide combinations used in the classic theranostic concept such as ^64^Cu/^67^Cu as a true matched pair or ^68^Ga/^177^Lu or ^68^Ga/^225^Ac as matched pairs, radiohybrid ligands do not use two radiometals, but combine a (radio)metal with a (radio)halogen instead.^2–4^ These ligands completely fulfil the radionuclide theranostic concept by allowing radiolabelling of the same molecule with two different radionuclides, thus generating an adapted definition of a true theranostic pair – a “radiohybrid pair”. When labelled with a radiohalogen, the other site is coordinated with the nonradioactive metal, and when labelled with a radiometal the other site bears the nonradioactive halogen like ^19^F or ^127^I.^4^

**Fig. 1:**
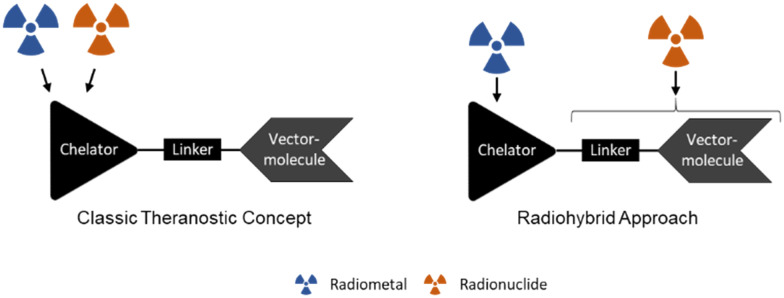
Comparison of the classic radionuclide theranostic concept and the radiohybrid approach showing different binding sites of the radionuclides.

The rationale behind this approach is to address the shortcomings of existing medical radionuclides, which are limited by their inherent constraints. The introduction of a radiohybrid approach aims to bridge this gap by providing a means to overcome the lack of suitable nuclides for the opposite nuclear medicine application. For example, a therapeutic analogue for fluorine-18 or a diagnostic counterpart for actinium-225 are unknown, but both could be combined using two different labelling moieties in the one molecule (Fig. 1). In this special case, both positions (complexing agent and halogen binding position) can be labelled independently of each other, thus ideally separating the respective radiolabelling process.^5^

**Fig. 2:**
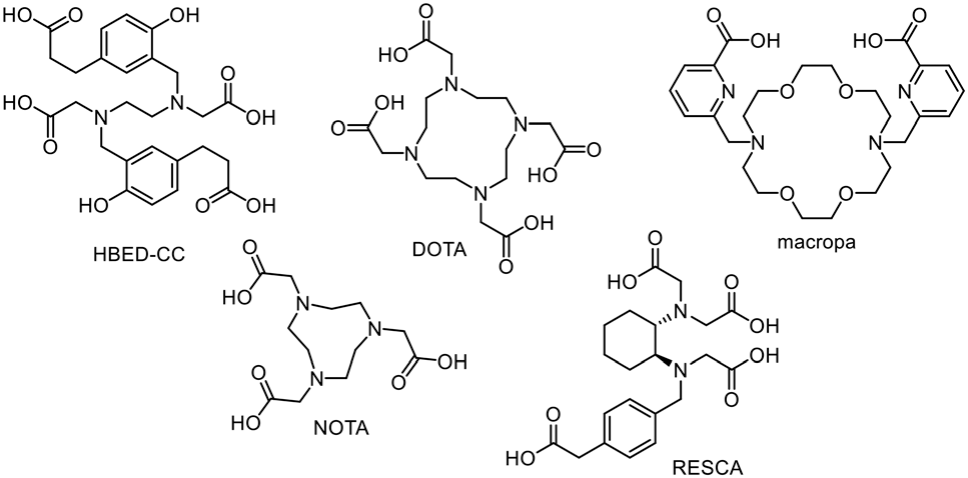
Cyclic and open-chain chelators commonly used in radiopharmacy and nuclear medicine.

The classic radionuclide theranostic concept^6^ (Fig. 1) involves the use of radiometals usually being complexed by a multidentate chelator (selection in Fig. 2). The radionuclides clinically used so far, such as ^43/44^Sc, ^68^Ga, ^90^Y, ^111^In and ^177^Lu, can be inserted in the same chelator, such as DOTA (2,2′,2′′,2′′′-(1,4,7,10-tetraazacyclododecane-1,4,7,10-tetrayl)tetraacetic acid). However, this may result in different coordination spheres and thus different chemical structures of the metal complexes.^7,8^ For example, a hexadentate complex is formed by the complexation of trivalent gallium with DOTA, while an octadentate complex is formed with trivalent lutetium.^9–11^ These “matched pairs” are in fact very similar, but often demonstrate non-identical pharmacological behaviour.^4,11,12^ In this case, [^68^Ga]Ga-DOTA-TATE is used for diagnosis and [^177^Lu]Lu-DOTA-TATE for targeted endoradiotherapy of neuroendocrine tumours but both radiotracers show pharmacokinetic differences.^9,13^ This is in contrast to rh-ligands, which are *per se* chemically identical and provide the same pharmacokinetic characteristics as “true matched pairs”.^5^ Notably, the combination of [^68^Ga]Ga-PSMA-11 (Illuccix, Locametz or isoPROtrace-11) with [^177^Lu]Lu-PSMA-617 (Pluvicto) is a prominent clinically established example using different chelators. HBED-CC (*N*,*N*′-bis-[2-hydroxy-5-(carboxyethyl)benzoyl]ethylenediamine-*N*,*N*′-diacetic acid) and DOTA are used, respectively, which significantly influences the pharmacological behaviour of the given radiotracer resulting in different distribution profiles of the tracers in the body.^10,11^ Even the use of PSMA-617 with DOTA as a chelator for both radionuclides shows a change in the *in vivo* kinetics.^10^ In this context, the introduction of the radiohybrid approach offers a promising solution to assure identical biodistribution behaviour. In contrast, the influence of the chelator plays a minor role like in the case of [^111^In]In-ibritumomab for SPECT and [^90^Y]Y-ibritumomab for therapy of non-Hodgkin's lymphoma as the behaviour is largely dependent on the macromolecular antibody.^14,15^

**Fig. 3:**
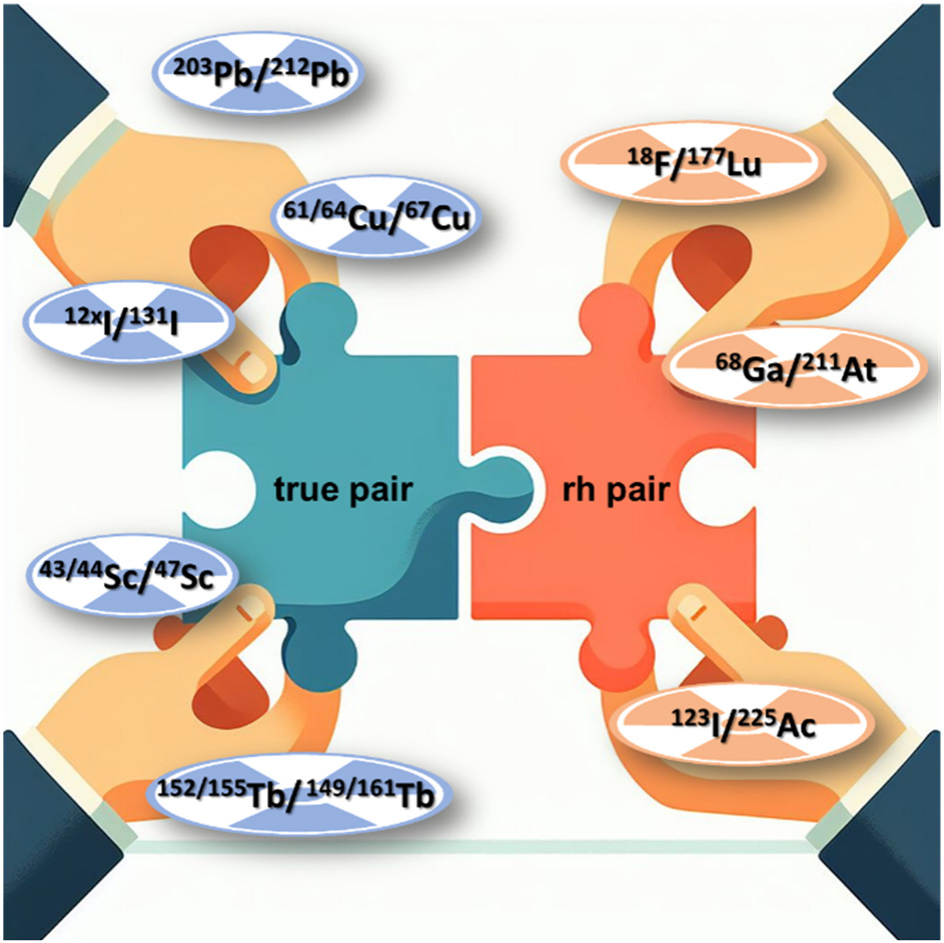
The radiohybrid approach adds new “rh pairs” to the number of “true matched pairs”.

Opposite to the classic concept, rh ligands contain an additional binding site for a covalently bound radiohalogen. This makes the combination of metals and non-metals easily possible without changing the chemical structure of the molecule. Consequently, the radiohybrid approach elegantly extends the limited number of matched pairs for radionuclide theranostic applications (Fig. 3),^7^ which opens up new possibilities for clinical applications.

### Fluorine-18 and/or gallium-68?

In principle, the use of radionuclides such as ^68^Ga and ^177^Lu represents a viable radionuclide theranostic strategy, as is the case with PSMA-I&T.^16^ However, the hybrid combination of ^18^F and ^177^Lu offers several advantages over gallium. ^18^F has a more comfortable half-life of 110 minutes, almost twice as long as ^68^Ga with a physical half-life of 68 minutes (Table 1). A longer handling in combination with imaging at later time-points could be favourable for providing more comprehensive data on the biodistribution and clearance of the radiopharmaceutical; *e.g.*, a particular advantage of combining ^18^F and ^177^Lu in one molecular structure for a more accurate dosimetric calculation.^8^ The lower positron energy of fluorine-18 results in a higher resolution and better quality of the PET images, with the advantage to even detect smaller metastases.^17^

**Table 1:** Comparison of radionuclides discussed in this review

Radionuclide	Production	Half-life	Energy
^18^F	^18^O(p,n)^18^F	110 min	0.65 MeV (β^+^)
^68^Ga	^68^Ge/^68^Ga generator	68 min	1.90 MeV (β^+^)
^123^I	^124^Xe(p,2n)^123^Cs → ^123^Xe → ^123^I	13.2 h	0.159 MeV (EC)
^177^Lu	^176^Lu(n,γ)^177^Lu	6.7 d	0.498 MeV (β^−^)
^211^At	^209^Bi(α,2n)^211^At	7.2 h	5.87 MeV (α)
^225^Ac	^227^Th decay	9.9 d	5.8 MeV (first α)

The production methods are also different. The fact that ^68^Ga is obtained by elution from a generator means that only limited activity can be achieved, but at least more frequently and cyclotron-independent.^18^ With ^18^F as a cyclotron-produced radionuclide, much higher activities can be obtained during production. This means that fluorine-18 can be produced on a large scale for significantly more patients.^19,20^

### Matched pair radiohalogen/radiometal complexes

**Fig. 4:**
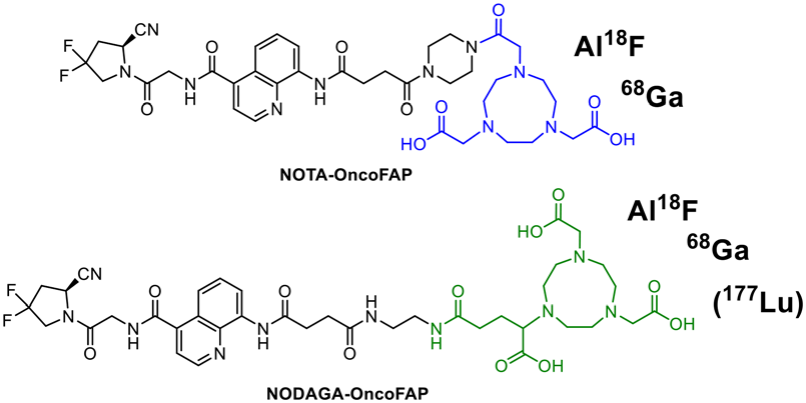
FAPα-conjugates for hybrid radiolabelling using radiometals and the Al^18^F technique.

Various attempts have been made in the past to combine a diagnostic radiohalogen (^18^F, ^12*x*^I) with a therapeutic radiometal. One of these is based on the Al^18^F approach, which is based on the chelating system for radiometal coordination.^21^ Advantageously, the aluminium–fluorine-bond is one of the strongest bonds and the labelling with [^18^F]fluoride works in acceptable yields in a short period of time in most cases without azeotropic drying. The NOTA chelator (2,2′,2′′-(1,4,7-triazacyclononane-1,4,7-triyl)triacetic acid) or open-chain chelators like RESCA 2,2′-(((1*S*,2*S*)-2-((carboxymethyl)(4-(carboxymethyl)benzyl)amino)cyclohexyl)azanediyl)diacetic acid or HBED-CC are required to stably bind the AlF moiety. One example of a successful dual labelling is presented in Fig. 4 for FAPα-radioconjugates realising the combination of the diagnostic radionuclides ^18^F/^68^Ga using the same precursor molecule.^22^

Radiolabelling with ^68^Ga delivered both radiotracers [^68^Ga]Ga-NOTA-OncoFAP and [^68^Ga]Ga-NODAGA-OncoFAP in high radiochemical yields (RCYs) of >88% in high molar activities (*A*_m_) of 26–39 GBq μmol^−1^ and radiochemical purities (RCPs) >99% in an automated radiosynthesis procedure (5–10 min, 95 °C). In contrast, radiolabelling with ^18^F (radiosynthesis time *ca.* 25 min, 95 °C) delivered [^18^F]AlF-NOTA-OncoFAP in 20% RCY (*A*_m_ = 3–9 GBq μmol^−1^), whereas [^18^F]AlF-NODAGA-OncoFAP was obtained in only 2% RCY (*A*_m_ = 0.4 GBq μmol^−1^). For radiolabelling with ^177^Lu, the chelator was changed to NODAGA leading to an altered radioconjugate. With this approach, a change in the pharmacological behaviour of the radioconjugates cannot be ruled out due to the different binding situations (AlF *vs.* radiometal) in the chelator.^22^

### Radiohybrid ligands

**Fig. 5:**
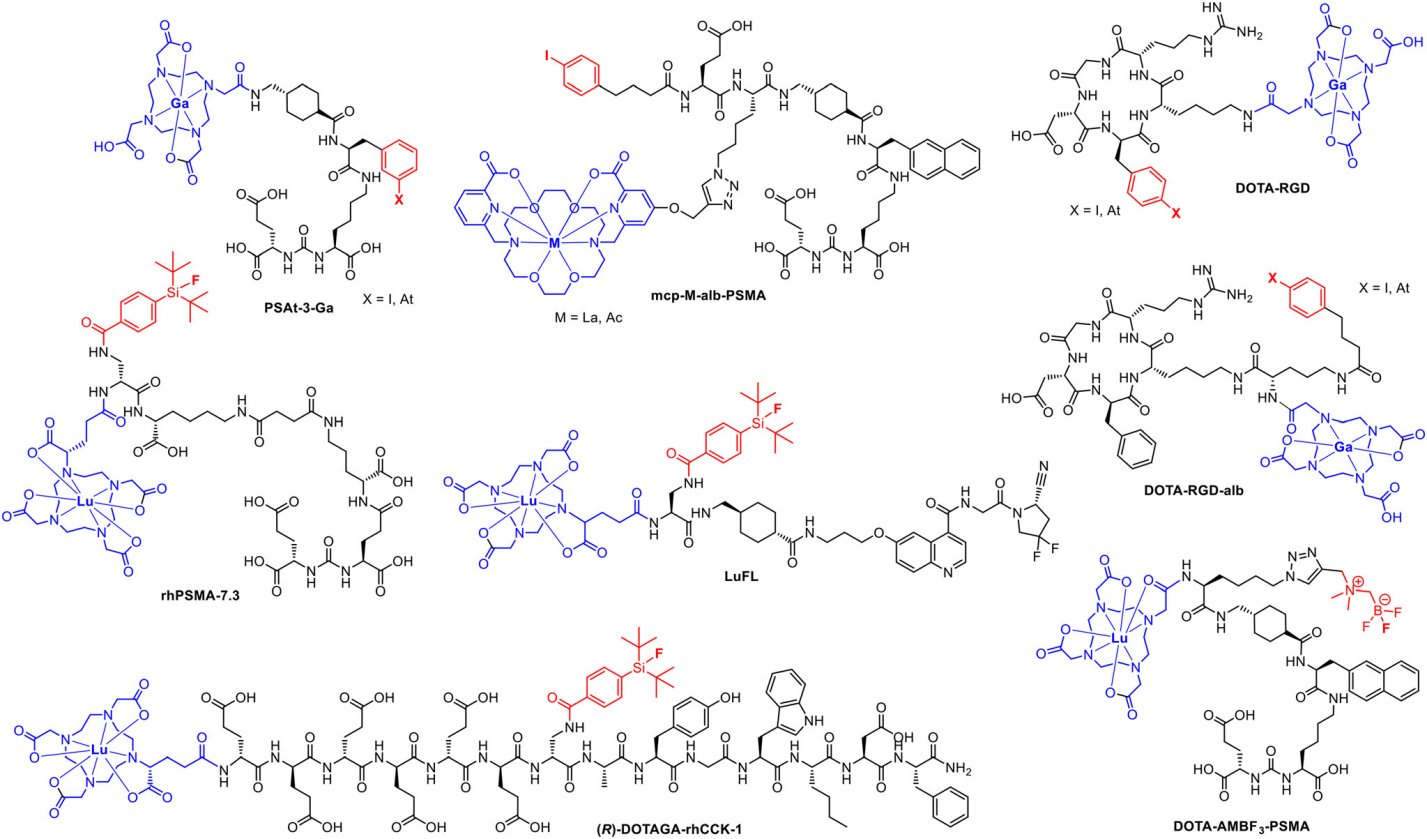
Overview over ligands and conjugates applied in the radiohybrid approach including the recently approved [^18^F]Ga-rhPSMA-7.3.

To date, only a limited number of radiohybrid conjugates are known (Fig. 5), including [^18^F]F-rhPSMA-7.3 (recently approved as Posluma; flotufolastat F18),^23^ LuFL, DOTAGA-rhCCK1, DOTA-RGD, mcp-M-alb-PSMA and DOTA-AMBF_3_-PSMA. All conjugates facilitate radiolabelling according to the radiohybrid approach to fulfil the theranostic concept. Most of the examples shown are based on the combination of ^18^F and ^177^Lu. One combination is based on the alpha emitter ^211^At with ^68^Ga (DOTA-RGD-alb) and one is based on ^225^Ac with ^123^I (mcp-M-alb-PSMA).

DOTA or DOTAGA serve as preferred chelators for the complexation of ^68^Ga or ^177^Lu in all the conjugates depicted in Fig. 5, except of the macropa-based mcp-M-alb-PSMA.^24^ In this example, macropa was used for the complexation of ^225^Ac to overcome obstacles with the *in vivo* stability of ^225^Ac–DOTA-complexes. Furthermore, ^18^F was introduced by the isotopic exchange reaction *via* the silicon-fluoride acceptor (SiFA) moiety or using the BF_3_ unit located in the periphery.^25^ The rh-approach can be applied to various targeting molecules such as prostate specific membrane antigen (PSMA) binders,^26–28^ the fibroblast activation protein inhibitor (FAPI),^8^ the cholecystokinin-2 receptor targeting minigastrin^17^ or the α_v_β_3_ integrin targeting cyclic peptide sequence RGD (arginine–glycine–aspartic acid).^29^ The easy transfer of the radiohybrid approach to many different biological targets makes it a comprehensive tool for various cancer entities.

### rhPSMA conjugates

The PSMA protein is overexpressed in malignant prostate tissue, whereas it is expressed only at low levels in healthy tissue. This makes PSMA an excellent biological target for diagnosis and treatment of prostate cancer.^30^ A high number of PSMA-targeting radioligands are known, including the diagnostic gold standard [^68^Ga]Ga-PSMA-11 and the therapeutic counterpart [^177^Lu]Lu-PSMA-617.^31^

In a first report in 2020, six different radiohybrid PSMA ligands (named as rhPSMA ligands of the rh-PSMA-7 series) were evaluated^32,33^ and compared with the commonly used ^18^F-labelled PSMA radioconjugates [^18^F]F-DCFPyL (piflufolastat) and [^18^F]F-PSMA-1007.^27^ All radiohybrid compounds contain the 4-(di-*tert*-butylfluorosilyl)benzoyl residue allowing the isotope exchange according to the SiFA route.^33^ Furthermore, different chelators DOTA, DOTAGA and NOTA-based TRAP were used for radiolabelling with ^68^Ga and ^177^Lu.^27^

The peptide structure with the PSMA-binder was prepared by solid-phase peptide synthesis *via* the Fmoc protecting group strategy. The 4-(di-*tert*-butylfluorosilyl)benzoyl moiety was introduced as supplemental fluorine binding site and DOTAGA anhydride as chelator for the radiometal.^27^ The labelling with ^177^Lu was carried out in a NaOAc buffer (pH 5.5) with 20–50 MBq [^177^Lu]LuCl_3_ at 90 °C for 30 minutes, achieving a RCP of over 99%.^4^ For the isotope exchange reaction of fluorine, aqueous [^18^F]fluoride was first dried using an anion exchange cartridge, eluted with K_2.2.2._/KOH, neutralised with oxalic acid and reacted with the precursor in anhydrous DMSO. The radiolabelling was completed after 5 minutes at room temperature and offers a RCY of approximately 60% after a convenient cartridge purification.^27^

**Fig. 6:**
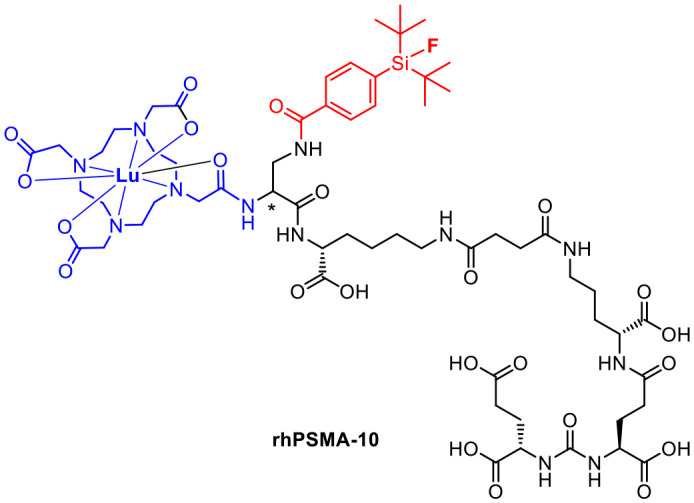
Molecular structure of the pharmacologically improved rhPSMA-10.

Compared to [^18^F]F-DCFPyL and [^18^F]F-PSMA-1007, rhPSMA-7 showed an improvement in internalisation and binding affinity.^27^ Consequently, rhPSMA-7.3 was selected as the lead compound on the basis of preclinical biodistribution data including a general lower uptake in liver and kidneys paired with a lower blood circulation and a high tumour uptake.^34–36^ The next generation has already been published as the successor. [^177^Lu]Lu-rhPSMA-10 (Fig. 6) is expected to have an even faster clearance from healthy tissue such as the kidneys. The charge was reduced by replacing DOTAGA by DOTA.^37^

### DOTA-AMBF_3_-PSMA

One advantage of the AMBF_3_ ((alkyldimethylammonio)methyl)-trifluoroborate group is that it can be radiolabelled quickly and under mild conditions with [^18^F]fluoride *via* isotope exchange. The fluorine binding site was introduced by binding AMBF_3_ to Fmoc-azidolysine using the Cu-catalyzed azide–alkyne click reaction.^38^ The carboxylic acid of the clicked compound was then converted to the NHS active ester and bound to PSMA-617-NH_2_ synthesized by solid phase peptide synthesis. The chelator was introduced as DOTA-NHS ester as well. Radiofluorination was performed by adding [^18^F]fluoride (74 GBq) eluted from an anion exchanger to the precursor (80 nmol) dissolved in aqueous pyridazine-HCl buffer solution (pH 2). The reaction was heated to 80 °C for 25 minutes, but the RCY was only about 1%. It is stated here that the reaction is still unoptimised and higher yields can be expected in the future. Labelling with ^177^Lu was performed by adding about 1 GBq [^177^Lu]LuCl_3_ to the precursor (10 nmol) dissolved in acetate buffer (pH 4.5). The solution was heated to 90 °C for 15 min and a radiochemical yield of 41% was obtained.^26^

Typically, significantly higher RCYs were reached with the SiFA method, which in turn favours the clinical application of SiFA. Additionally, the relatively mild SiFA method is capable of labelling temperature sensitive compounds, whereas the BF_3_ method invariably necessitates heating. However, with AMBF_3_, an [^18^F]F^−^/[^18^O]H_2_O-mixture can be used for radiolabelling, obviating the necessity for an additional drying step.^25^ Both methods have their respective advantages, but the SiFA method appears to be superior.

### PSAt-3-Ga

Radioconjugates containing ^211^At and the iodine isotopes ^123^I and ^131^I, have been extensively studied for their potential use in imaging and therapy. Due to their shared chemical similarities as halogens, astatine and iodine exhibit similar behaviour in biological systems and have the ability to form compounds with similar properties.^39^ Isotopes of both elements are suitable for medical applications, including targeted radionuclide therapy and diagnostic imaging. While iodine radioisotopes are more commonly used in clinical practice, ^211^At is gaining recognition for its potential therapeutic properties, especially in targeted alpha therapy.^40^ The on-site cyclotron production of ^211^At (^209^Bi(α,2n)^211^At) could be one advantage of ^211^At over other alpha-emitters like ^225^Ac. Furthermore, At can chemically be handled as a halogen. Thus, radiolabelling is achievable by electrophilic destannylation in the same manner as for (radio)iodine. However, it should be noted that the carbon–astatine-bond is susceptible to deastatination due to the lower energy of the At–C-bond, as shown by *in vivo* data.^41^

**Fig. 7:**
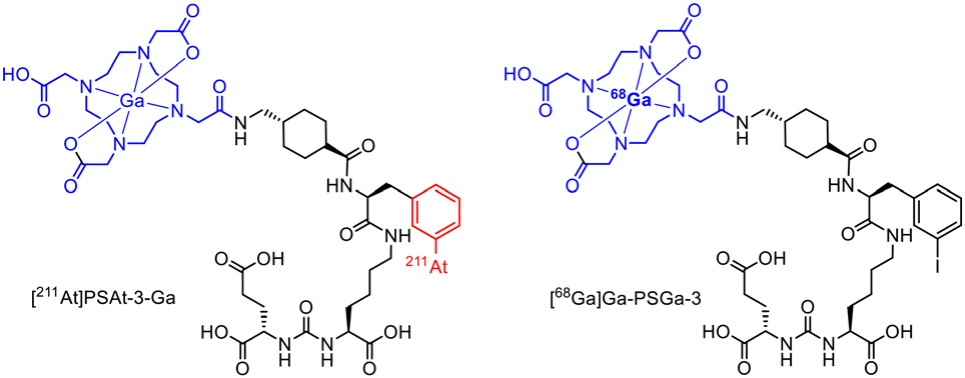
Molecule structures of [^211^At]PSAt-3-Ga and [^68^Ga]Ga-PSGa-3.^28^

Astatination reactions were commonly executed as electrophilic aromatic substitutions (S_E_Ar) requiring an electrophilic leaving group such as boronyl, stannyl or silyl. For this purpose, the desired trimethylsilyl precursor was synthesized using a solid phase peptide synthesis using an Fmoc-protecting group strategy. The binding site for the astatine labelling was attached using 3-(trimethylsilyl)phenylalanine, which was prepared beforehand. Radiolabelling with astatine was carried out under oxidative conditions by adding the precursor, *N*-chlorosuccinimide and TFA to [^211^At]At^−^. After the labelling at 70 °C for 10 minutes, the complexation with ^nat^Ga(NO)_3_ was performed in NaOAc-buffer (pH 5.5) to obtain the final ^211^At-radioconjugate [^211^At]PSAt-3-Ga (Fig. 7) with a RCY of 35% after cartridge purification. To obtain the diagnostic counterpart, the iodine-containing precursor was used for radiolabelling with [^68^Ga]Ga^3+^ (pH 4.0, 95 °C, 5 min), whereby a radiochemical conversion (RCC) of 98% was achieved for [^68^Ga]Ga-PSGa-3 (Fig. 7). Compared to PSMA-617, PSAt-3-Ga has a 50% lower tumour residence time, which is attributed to the irradiation of tumour cells with alpha particles and their subsequent death. However, the compound also shows a slight deastatisation with an accumulation in the thyroid gland of around 4% ID g^−1^.^28^

### LuFL

The fibroblast activation protein (FAP) is known as a versatile target present in a variety of malignancies with high expression in or close to tumors but negligible expression in healthy tissues. It is a membrane-bound protease with high expression in the tumor microenvironment of many solid cancer entities due to its presence on cancer-associated fibroblasts,^42,43^ a central component of the tumor microenvironment in primary and metastatic tumors.^44^ DOTAGA and SiFA represent the basis for radiolabelling in this radiohybrid example to prepare radioconjugates targeting FAP. The basic precursor was synthesized over a 20-step synthesis. For radiofluorination, the precursor was first coordinated with lutetium by adding ^nat^LuCl_3_ in NaOAc (pH 5) at 95 °C for 2 h. After purification by HPLC, the radiofluorination was carried out for 5 minutes at ambient temperature by adding oxalic acid and the ^nat^Lu-containing precursor to previously dried [^18^F]F^−^ eluted with K_2.2.2_ achieving a RCY ranging from 18 to 62% after cartridge purification. For the labelling with ^177^Lu, the unmodified precursor was dissolved in NaOAc, [^177^Lu]LuCl_3_ was added and the mixture was heated at 90 °C for 10 minutes reaching an RCY of >99% (*A*_m_ = 9.5–37.1 GBq μmol^−1^).^7^ This combination even enables a kit-like production of both the ^18^F- and the ^177^Lu-radioconjugate. LuFL shows higher cellular uptake, binding affinity, tumour uptake and retention time *in vivo* compared to FAPI04.^7^

### DOTA-RGD

**Fig. 8:**
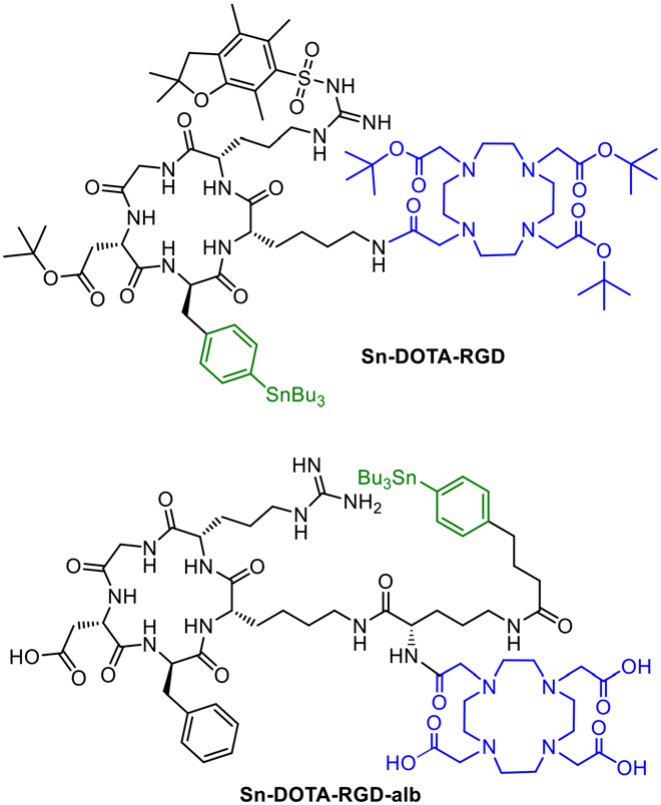
Molecule structures of the ^*t*^Bu-protected RGD-stannyl precursor Sn-DOTA-RGD and the stannyl-precursor Sn-DOTA-RGD-alb with albumin binder.

A *cyclo*-RGD peptide containing an additional phenylalanine moiety was used as the binding unit to target α_v_β_3_ integrin.^29^ The phenyl group facilitates introduction of radioiodine and astatine *via* electrophilic aromatic substitution. Radiohybrid conjugates based on the RGD peptide were synthesized by solid phase peptide synthesis using a Fmoc protecting group strategy. In this case, phenylalanine of the original RGD peptide was exchanged by 4-iodophenylalanine. DOTA was used as chelator and introduced as the tris-*tert*-butyl protected motif. To allow for the radiohalogenation under electrophilic aromatic substitution conditions, the iodo-containing peptide was stannylated by a tin–iodine exchange. Radiolabelling with ^211^At was carried out as electrophilic aromatic substitution using *N*-chlorosuccinimide (NCS)/acetic acid as the oxidizing reagent. The tin-precursor was dissolved in acetonitrile, the [^211^At]At^−^ solution and NCS were added, and the reaction mixture was heated to 80 °C for 15 minutes. The final deprotection was carried out with TFA prior to labelling with ^nat^Ga, followed by HPLC purification to obtain the ^211^At-radioconjugate in 16% RCY.^29^ In this case, the protected precursor Sn-DOTA-RGD (Fig. 8) was used for radiolabelling to avoid loss of the acid-labile stannyl group and subsequent radioiodination *via* tin–iodine exchange, which was shown to fail with the deprotected peptide precursor.^29^

Due to its longer half-life of 78 hours, ^67^Ga (suitable for SPECT) was used for radiolabelling instead of ^68^Ga (PET). Therefore, the precursor with nonradioactive iodine was dissolved in ammonium acetate buffer (pH 5.0) and [^67^Ga]GaCl_3_ (1.6 MBq) was added and reacted at 80 °C for 5 minutes. A RCY of 97% was obtained after purification using RP-HPLC.^29^

Biodistribution experiments in tumour-bearing mice showed a similar distribution pattern of the radioiodine ([^nat^Ga,^125^I]I-DOTA-RGD) and astatine ([^nat^Ga,^211^At]At-DOTA-RGD) radioconjugates.^29^ Due to the chemical similarity of iodine and astatine, this radiohybrid approach can be readily transferred to the ^68^Ga/^211^At combination.

To improve the pharmacokinetics of the radioconjugate, the structure was modified to include the albumin binding 4-(iodophenyl)butyrate group.^45^ For this purpose, the albumin binder 4-(iodophenyl)butyrate was additionally attached, allowing radiolabelling with ^125^I and ^211^At on this motif instead of the previously used phenylalanine of the RGD moiety. As a result, a prolonged blood clearance together with an enhanced tumour accumulation and retention was observed with the altered ^211^At-radioconjugate including the albumin binder. Tumour growth was significantly inhibited in tumour-bearing mice.^45^ The respective stannylated precursor Sn-DOTA-RGD-alb (Fig. 8) without protecting groups was used for radiolabelling. ^67^Ga was used as a SPECT nuclide.

### rhCCK conjugates

**Fig. 9:**
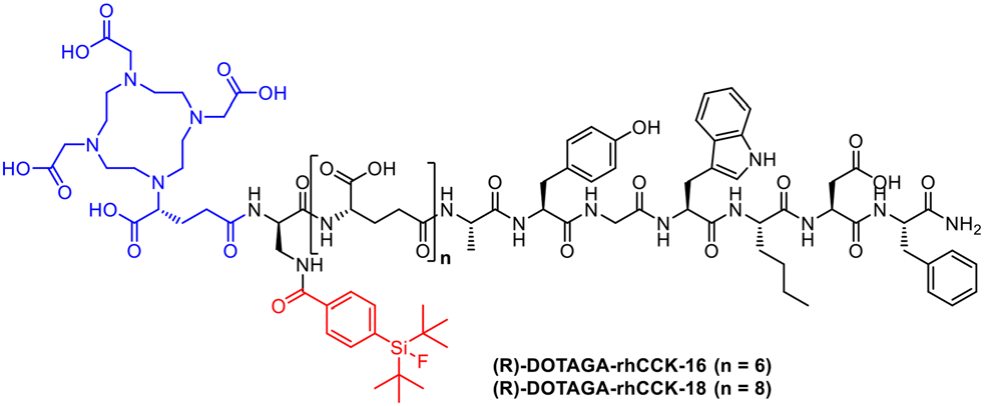
Molecular structures of (*R*)-DOTAGA-rhCCK-16 and -18.

The peptidic minigastrin derivative PP-F11N to target CCK-2R for addressing thyroid cancer was previously developed.^46^ (*R*)-DOTAGA-rhCCK-1 was initially designed on this basis using DOTA as chelator and SiFA for ^18^F-introduction as part of this radiohybrid approach.^17^ In order to optimize elevated kidney retention, two new radioconjugates DOTAGA-rhCCK-16 and DOTAGA-rhCCK-18 (Fig. 9) were investigated.^47^ Interestingly, the insertion of the SiFA unit resulted in a higher receptor affinity as well as internalisation due to the higher binding to human serum albumin.

The peptide backbone was synthesized by solid phase peptide synthesis using the Fmoc protecting group strategy.^17^ Radiolabelling with ^177^Lu for both conjugates was performed at 90 °C for 15 min in NaOAc-buffered HCl (pH 5.5) with quantitative RCYs and RCP with >95%. The radiofluorination of [^nat^Lu]Lu-DOTA-rhCCK-18 was performed at 60 °C for 5 min using previously dried [^18^F]fluoride *via* an isotopic exchange reaction at the SiFA site, followed by a cartridge purification with RCYs between 10 and 30% as well as RCPs > 95%.^42^*In vivo*, F-[^177^Lu]Lu-DOTA-rhCCK-18 shows very high tumour accumulation after only 1 h. A better detection rate is predicted for [^18^F]F-Lu-DOTA-rhCCK-18 compared to other CCK-2R or SSTR2-targeting compounds, suggesting a clinical translation.^47^

### mcp-M-alb-PSMA

The combination of radioiodine for SPECT and ^225^Ac for targeted alpha therapy with a PSMA-binding vector based on PSMA-617 represents a further enhancement of the radiohybrid approach. Macropa was used as a chelating system for the Ac coordination due to the formation of more stable complexes in contrast to DOTA.^48^ Concurrently, the albumin binder in the form of iodophenylbutyric acid was used to introduce the SPECT nuclide ^123^I through using a tin precursor strategy.^49^ The new radiohybrid pair of ^123^I and ^225^Ac was thus created. As no stable isotope of actinium is known, nonradioactive lanthanum can be used as a surrogate.^50^ The aforementioned difference of gallium and lutetium in the metal complexation plays a minor role for actinium and lanthanum as both have the same coordination properties and similar ion radii.^51^

Two albumin-binding radioconjugates containing the 4-iodophenyl butyrate moiety and macropa as chelator for ^225^Ac were developed. In this special case, the albumin binder was converted to allow radiolabelling with radioiodine. The PSMA-617-derivatised vector molecule was synthesized in solution using an Fmoc strategy.^52^ Macropa was connected to an azidolysine moiety *via* the Cu-catalysed azide–alkyne click reaction. For radioiodination, the iodine binding site is introduced *via* an 4-(trimethylstannyl)phenylbutyric acid-PNP active ester or, in the nonradioactive case, 4-(iodo)phenylbutyric acid. Radioiodination is carried out as an electrophilic aromatic substitution with iodogen as the oxidising reagent. To ensure radioiodination with ^123^I, the stannyl-precursor was dissolved in dimethyl sulfoxide and treated with [^123^I]I^−^ solution (up to 1 GBq) in a iodogen reaction tube and reacted at room temperature for 20 minutes. A RCY of approximately 10% was obtained.^31^ Radiolabelling with ^225^Ac was performed by dissolving the iodophenylbutyrate-precursor in an ammonium acetate buffer (pH 6) and adding [^225^Ac]AcCl_3_ at room temperature for 15 minutes with a RCY > 99%.^48^

A significant difference is that two different precursor molecules are required in this example for the iodination or complexation of actinium, which is not the case when SiFA in combination with chelator are used. Nevertheless, the resulting radioconjugates ultimately have the same properties.

## Conclusion and outlook

Within the theranostic concept, the radiohybrid approach offers a new opportunity to combine radionuclides that previously had no therapeutic or diagnostic counterpart. For the first time, a true theranostic approach is possible for the PET nuclide ^18^F, which can be now combined with, *e.g.*, ^177^Lu, ^225^Ac or other therapeutic radionuclides. To achieve the radiohybrid approach, existing chelator-based radiopharmaceuticals could be extended, *e.g.*, by a SiFA unit or existing iodinated albumin binders could be used. However, the introduction of a SiFA binding site can lead to an increased lipophilicity which can cause an alteration of the pharmacological behaviour.^7^ In the case of the rhCCK conjugates, a higher tumour uptake was observed. As a drawback, isotopic labelling can also lead to ^18^F-radioconjugates with reduced molar activity.

An advantage of iodinated radioconjugates over fluorine isotope exchange compounds is that significantly higher molar activities can be achieved due to the use of precursors with leaving groups. The use of extra precursors for the radioiodine labelling and an additional purification step could be seen as a drawback here. ^211^At tracers must be studied preclinically with particular care to avoid deastatisation^53^ due to the relatively weak astatine carbon bond.

**Fig. 10:**
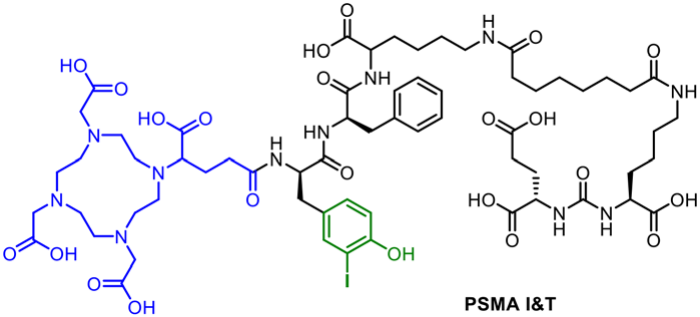
Molecular structure of PSMA I&T with DOTAGA chelating unit (blue) and the possibility for a radioiodine labelling (green).

The radiohybrid approach is, like all other conventional radiolabelling methodologies, usually limited to radionuclides of elements which also have a nonradioactive isotope.^27^ Astatine-211 and actinium-225 are exceptions. The use of iodine and lanthanum,^54,55^ respectively, as nonradioactive surrogates for analytical characterisation and for identification is a good compromise due to their similar chemical properties. The transfer of the radiohybrid approach within the theranostic concept is conceivable for all compounds and conjugates containing a halogen on the periphery of the molecule and a chelating system such as PSMA I&T^16^ (Fig. 10). In this case, the iodinated tyrosyl residue could provide the combination of radioiodine and ^177^Lu. However, it should be noted that the body's deiodination enzymes could lead to deiodination processes.^56^

**Fig. 11:**
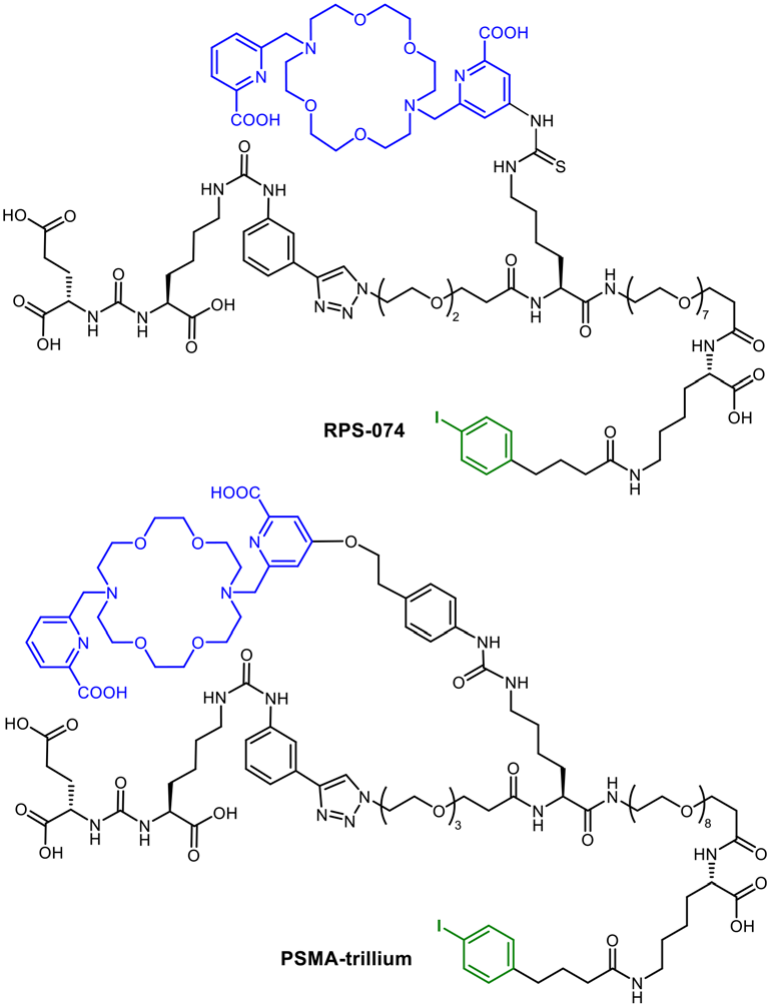
Molecular structures of RPS-074 and PSMA-trillium with macropa chelator (blue) and the possibility for labelling with radioiodine (green) using the albumin binder.

RPS-074^57^ and PSMA-trillium^58^ (Fig. 11) are other examples for a possible transfer of the radiohybrid concept. As realized for mcp-M-alb-PSMA with ^225^Ac and ^123^I, the albumin binders in RPS-074 and PSMA-trillium are eligible to be radioiodinated in same way. However, in the clinical trial (NCT06217822), [^111^In]In-PSMA-trillium containing the DOTA chelator was used as the SPECT-diagnostic counterpart and [^225^Ac]Ac-PSMA-trillium with a macropa chelator was used for dose escalation studies.

In comparison to the classic theranostic concept, it could be easier to translate compounds into the clinic, as the same biodistribution for patient application will bring advances in the combination of diagnosis and therapy. The inclusion of an additional binding site for a covalently bound radiohalogen in radiohybrid ligands opens up the possibility of combining metals and non-metals. Without altering the molecule pharmacologically, a significant advance in oncology research and clinical application is achieved. This was demonstrated successfully by SiFA-containing compounds where the introduction of the fluorine group does have a significant effect on the pharmacokinetics as could be shown with LuFL. One potential disadvantage is the increased effort required for the precursor synthesis. However, this flexibility enables the creation of innovative radiopharmaceutical compounds that can be targeted towards specific oncological sites, the amplification of radionuclides without a diagnostic or therapeutic counterpart to perform both diagnostic and therapeutic functions in a single (radio)conjugate. The integration of metals and radiohalogens in a single ligand as the basis of the radiohybrid approach thus represents a significant step forward in the further development of precise and individualised medical applications in nuclear medicinal research and clinical practice.

## Data availability

No primary research results, software or code have been included and no new data were generated or analysed as part of this review.

## Author contributions

T. Krönke, C. Mamat: methodology, investigation, visualization, writing – original draft. T. Krönke, C. Mamat: review & editing. K. Kopka: supervision & review. All authors have read and agreed to the published version of the manuscript.

## Conflicts of interest

There are no conflicts to declare.
